# Benchmarking of RNA-sequencing analysis workflows using whole-transcriptome RT-qPCR expression data

**DOI:** 10.1038/s41598-017-01617-3

**Published:** 2017-05-08

**Authors:** Celine Everaert, Manuel Luypaert, Jesper L. V. Maag, Quek Xiu Cheng, Marcel E. Dinger, Jan Hellemans, Pieter Mestdagh

**Affiliations:** 1 Center for Medical Genetics, Ghent University, Ghent, Belgium; 2 Cancer Research Institute Ghent, Ghent University, Ghent, Belgium; 3 Bioinformatics Institute Ghent N2N, Ghent University, Ghent, Belgium; 4 Biogazelle, Ghent, Belgium; 5 Kinghorn Cancer Center, Sydney, Australia

## Abstract

RNA-sequencing has become the gold standard for whole-transcriptome gene expression quantification. Multiple algorithms have been developed to derive gene counts from sequencing reads. While a number of benchmarking studies have been conducted, the question remains how individual methods perform at accurately quantifying gene expression levels from RNA-sequencing reads. We performed an independent benchmarking study using RNA-sequencing data from the well established MAQCA and MAQCB reference samples. RNA-sequencing reads were processed using five workflows (Tophat-HTSeq, Tophat-Cufflinks, STAR-HTSeq, Kallisto and Salmon) and resulting gene expression measurements were compared to expression data generated by wet-lab validated qPCR assays for all protein coding genes. All methods showed high gene expression correlations with qPCR data. When comparing gene expression fold changes between MAQCA and MAQCB samples, about 85% of the genes showed consistent results between RNA-sequencing and qPCR data. Of note, each method revealed a small but specific gene set with inconsistent expression measurements. A significant proportion of these method-specific inconsistent genes were reproducibly identified in independent datasets. These genes were typically smaller, had fewer exons, and were lower expressed compared to genes with consistent expression measurements. We propose that careful validation is warranted when evaluating RNA-seq based expression profiles for this specific gene set.

## Introduction

Due to the drop in cost of massively parallel sequencing, RNA-sequencing (RNA-seq) has become a viable alternative to gene expression microarrays^1^. Nowadays, RNA-seq is generally considered the gold standard for whole transcriptome gene expression quantification, not only in research but also for clinical applications. Compared to microarrays, RNA-seq has several major advantages. First, no prior knowledge about the content of the transcriptome is required, providing an unbiased view on the ensemble of transcripts in a sample and the possibility of evaluating allelic expression. Second, RNA-seq enables a much more detailed analysis of alternative splicing events. While certain microarray platforms can be used to study alternative splicing^2^, this is typically limited to known isoforms and occurs at much lower resolution. Finally, RNA-seq gene expression measurements tend to cover a much broader dynamic range and can be more sensitive compared to microarrays^3, 4^. Nevertheless, the field of RNA-seq still faces many challenges, especially in terms of data processing and analyses. In contrast to the microarray field, where data processing converged over the years into a well-defined set of broadly accepted workflows, the number of RNA-seq data processing workflows is still increasing, with none accepted as the standard so far. RNA-seq data processing workflows typically come in two different flavours. First, there are methods that align reads directly to a reference genome, followed by quantification of mapped reads (e.g. Tophat-Cufflinks^5^, Tophat-HTSeq^6, 7^ and STAR-HTSeq^7, 8^). Secondly, there are the so-called pseudoalignment methods (e.g. Salmon^9^ and Kallisto^10^) that break up reads into k-mers before assigning them to transcripts. This results in a substantial gain in speed compared to the alignment based workflows. The workflows also differ in how they estimate expression abundance, with some enabling quantification on transcript level (i.e. Cufflinks, Salmon and Kallisto) while others are restricted to gene level quantification.

Studies benchmarking RNA-seq processing workflows typically rely on simulated RNA-seq datasets or RT-qPCR data for just a few hundred genes^10–12^. Often, these studies focus their analysis on evaluating absolute quantification performance (i.e. gene expression correlation between RNA-seq and RT-qPCR data) without assessing relative quantification performance (i.e. differential gene expression correlation). Still, the latter is what most RNA-seq studies are aiming for. Recently, Teng and colleagues developed a series of performance parameters to evaluate RNA-seq quantification workflows^13^. Using both matching microarray data and simulated RNA-seq data, they concluded that the performance of the various workflows was comparable but poor.

Here, we compared RNA-sequencing data, processed using five workflows with expression data generated by wet-lab validated qPCR assays for 18 080 protein-coding genes. We decided to include workflows representative for the two major methodologies available today (i.e. pseudoalligment and alignment-based methods). For the alignment based methodologies, frequently used pipelines like Star/Tophat-HTSeq and Tophat-Cufflinks were selected whereas for the pseudo-alignment algorithms we included Salmon and Kallisto. The samples that were applied for this study are the well-characterized MAQC-I RNA-samples MAQCA (Universal Human Reference RNA, pool of 10 cell lines) and MAQCB (Human Brain Reference RNA)^14^. RT-qPCR is still considered the method of choice for validation of gene expression data obtained by high-throughput profiling platforms. We therefore reasoned that a transcriptome-wide RT-qPCR dataset would serve as a solid benchmark to assess the accuracy of the selected RNA-seq processing workflows. In addition, we provide an analysis framework that can be applied to other workflows not included in this study. While this is not the first study to compare RNA-seq data with transcriptome-wide qPCR data, the analyses presented here are more comprehensive compared to other studies.

## Results

### Aligning qPCR and RNA-seq datasets

Every assay included in the whole-transcriptome qPCR dataset detects a specific subset of transcripts that contribute proportionally to the gene-level Cq-value. In order to apply these as a benchmark for RNA-seq based gene expression values, we aligned transcripts detected by qPCR with transcripts considered for RNA-seq based gene expression quantification. For the transcript based workflows (Cufflinks, Kallisto and Salmon), we calculated the gene level TPM values by aggregating transcript-level TPM-values of those transcripts detected by the respective qPCR assays. For Tophat-HTSeq and Star-HTSeq, gene level counts were converted to gene-level TPM values. First, genes were filtered based on a minimal expression of 0.1 TPM in all samples and replicates, to avoid the bias for low expressed genes. This resulted in the selection of 13 045 and 13 309 genes for RNA-seq dataset 1 and 2 respectively. The mean expression across replicates was calculated and used for further analysis.

### Expression correlation

**Figure 1:**
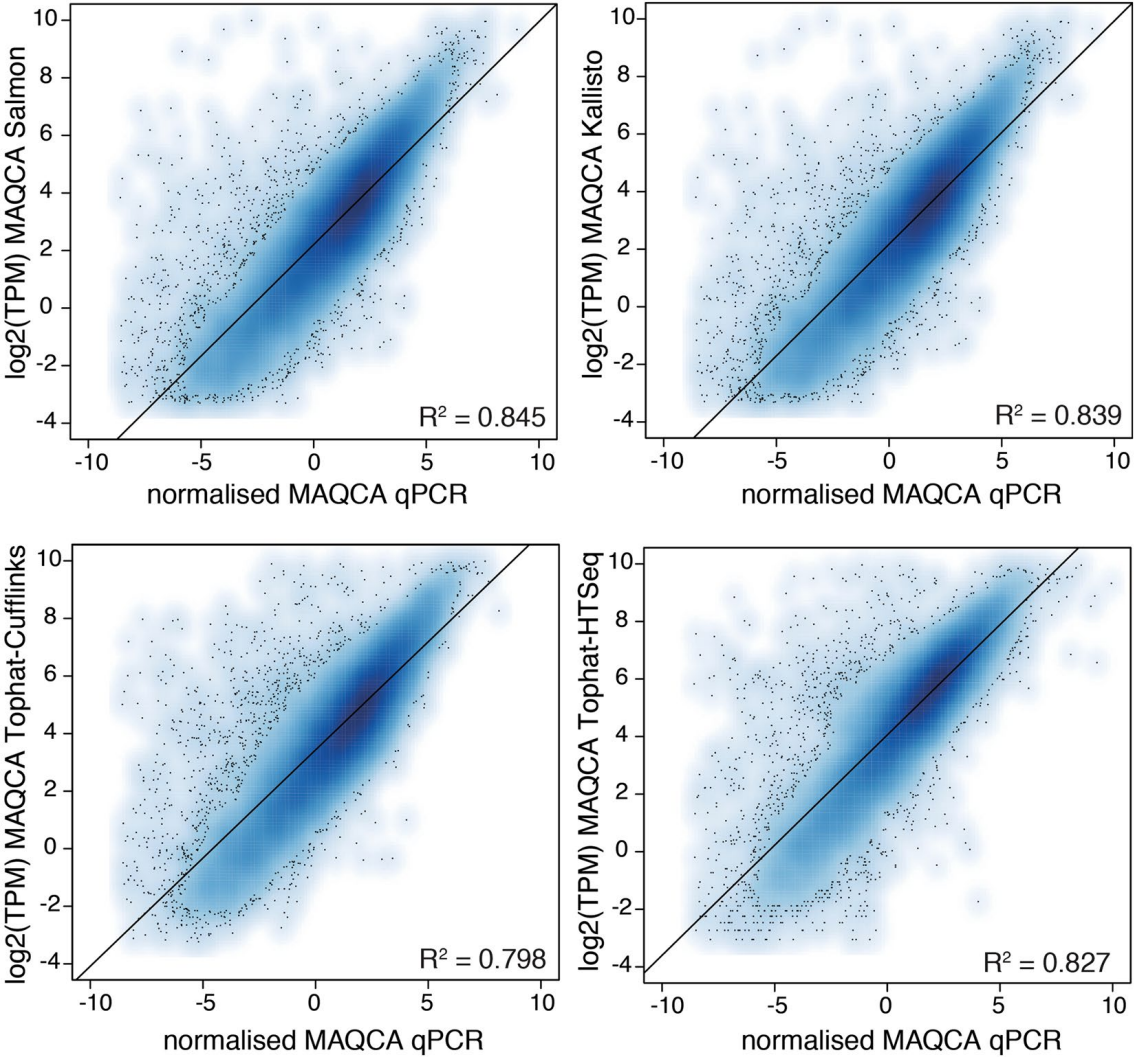
Gene expression correlation between RT-qPCR and RNA-seq data. The Pearson correlation coefficients and linear regression line are indicated. Results are based on RNA-seq data from dataset 1.

**Figure 2:**
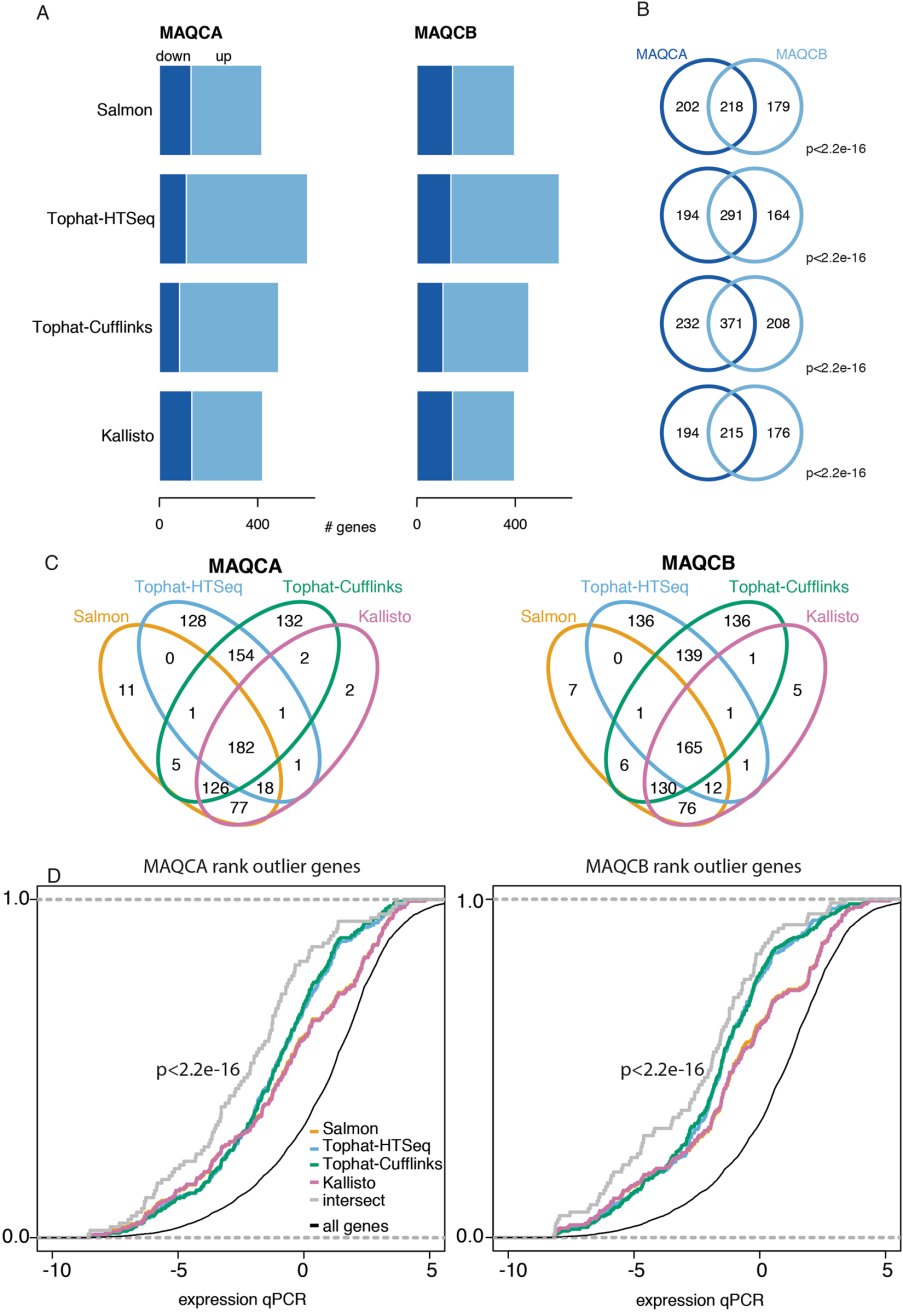
The overlap of the rank outlier genes between samples (MAQCA and MAQCB) and workflows is significant. (**A**) The number of genes with an (absolute) rank shift of more than 5000 are indicated. Genes marked as down have a higher expression rank in RT-qPCR, genes marked as up have a higher expression rank in RNA-seq. (**B**) The overlap of genes with an absolute rank shift of more than 5000 between MAQCA and MAQCB is significant for each workflow (Fisher exact test) (**C**) The overlap of the genes with an absolute rank shift of more than 5000 between the different workflows is significant (Super exact test). (**D**) Genes with an absolute rank shift of more than 5000 have an overall lower expression. The Kolmogorov-Smirnov p-value for the intersection of rank outlier genes between methods is shown. Results are based on RNA-seq data from dataset 1.

To evaluate concordance in gene expression intensities between RNA-seq and qPCR, we first calculated expression correlation between normalized RT-qPCR Cq-values and log transformed RNA-seq expression values. Overall, high expression correlations were observed between RNA-seq and qPCR expression intensities for all workflows (Pearson correlation, Salmon R^2^ = 0.845, Kallisto R^2^ = 0.839, Tophat-Cufflinks R^2^ = 0.798, Tophat-HTSeq R^2^ = 0.827, Star-HTseq R^2^ = 0.821) (Fig. 1, Supplemental Fig. 1a). Comparing expression values between Tophat-HTSeq and Star-HTSeq revealed almost identical results (R^2^ = 0.994, Supplemental Fig. 1b) suggesting little impact of the mapping algorithm on quantification. We therefore decided to only consider Tophat-HTSeq for further analysis. In order to further study discrepancies in gene expression correlation, we first transformed TPM and normalized Cq-values to gene expression ranks (Supplemental Figs 1c and 2) and calculated the difference in rank between RNA-seq and qPCR. Outlier genes were defined as genes with an absolute rank difference of more than 5000 (further referred to as rank outlier genes) (Fig. 2A). The average number of rank outlier genes ranged from 407 (Salmon) to 591 (Tophat-HTSeq) and the majority of these had higher expression ranks in RNA-seq data (i.e. higher expressed in RNA-seq data), irrespective of the workflow. Rank outlier genes for MAQCA significantly overlapped with rank outlier genes for MAQCB for each of the workflows (Fig. 2B, Fisher Exact test, p < 1.10^−10^). Also between workflows, a significant overlap was observed (Fig. 2C and Supplemental Fig. 3, Super Exact Test, p values < 1.10^−10^). These observations were confirmed in both datasets (Supplemental Figs 4–6) and point to systematic discrepancies between quantification technologies (i.e. qPCR and RNA-seq) rather than workflows. Still, a number of workflow-specific rank outlier genes were identified (Fig. 2B). The rank outlier genes are characterized by a significantly lower RT-qPCR expression value (Fig. 2D, Kolmogorov-Smirnov, p < 1.10^−10^), explaining at least part of the observed rank difference. Similar results were obtained in the second dataset (Supplemental Fig. 6).

### Fold change correlation

**Figure 3:**
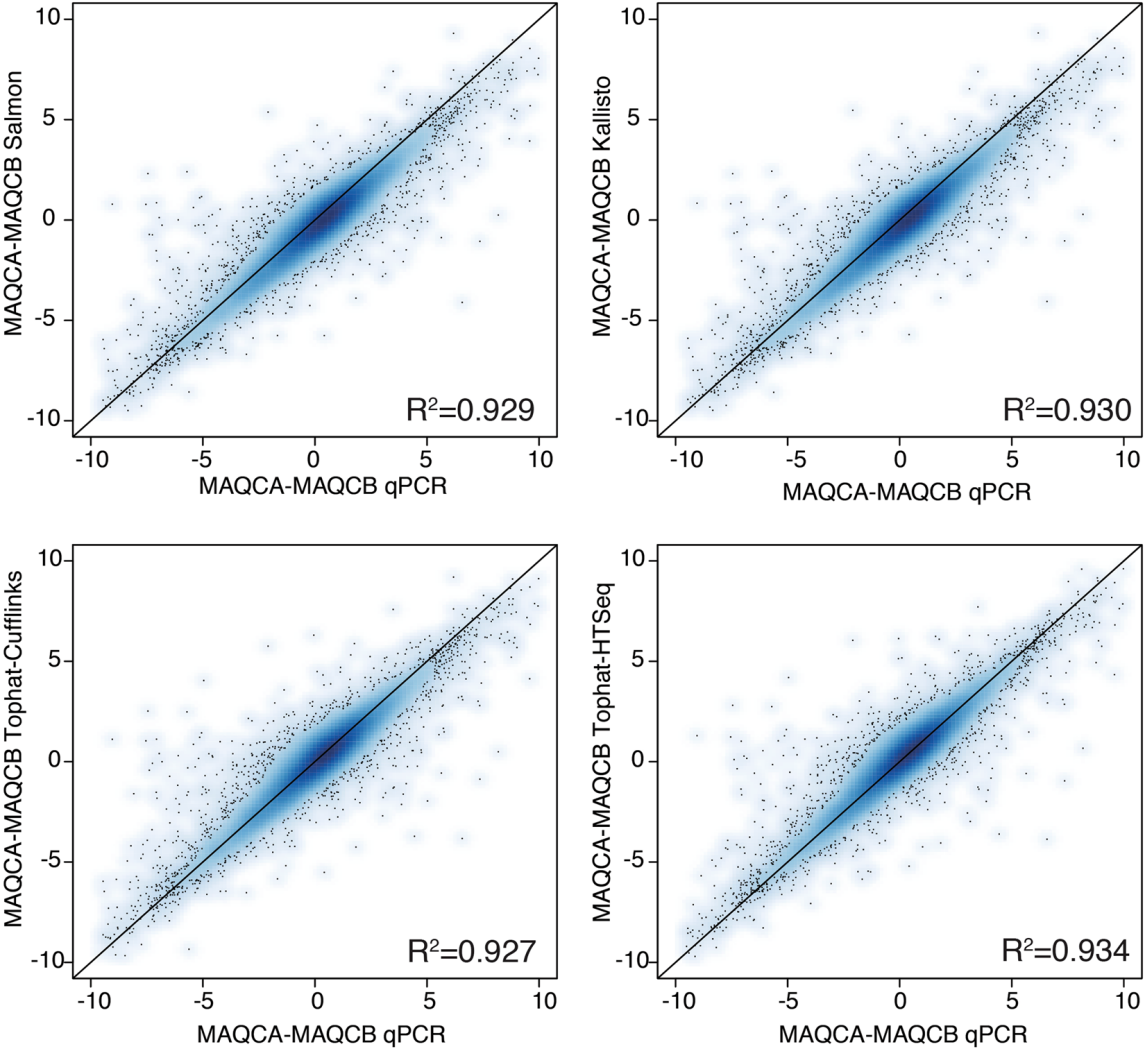
High fold change correlation between RT-qPCR and RNA-seq data for each workflow. The correlation of the fold changes was calculated by the Pearson correlation coefficient. Results are based on RNA-seq data from dataset 1.

As RNA-sequencing and qPCR produce relative gene expression measures, comparing gene expression differences between samples is the most relevant approach to benchmark RNA-seq quantification workflows. To this end, we calculated gene expression fold changes between MAQCA and MAQCB and evaluated fold change correlations between RNA-seq and qPCR. High fold change correlations were observed for each workflow (Fig. 3 and Supplemental Fig. 1d, Pearson, Salmon R^2^ = 0.929, Kallisto R^2^ = 0.930, Tophat-Cufflinks R^2^ = 0.927, Tophat-HTSeq R^2^ = 0.934, Star-HTseq R^2^ = 0.933) suggesting an overall high concordance between RNA-seq and qPCR with nearly identical performance for the individual workflows. As for the expression ranks, the fold changes obtained with Tophat-HTSeq and Star-HTSeq were highly identical (Supplemental Fig. 2f, R^2^ = 0.996), suggesting that the mapping algorithm does not effect fold change calculations between samples.

**Figure 4:**
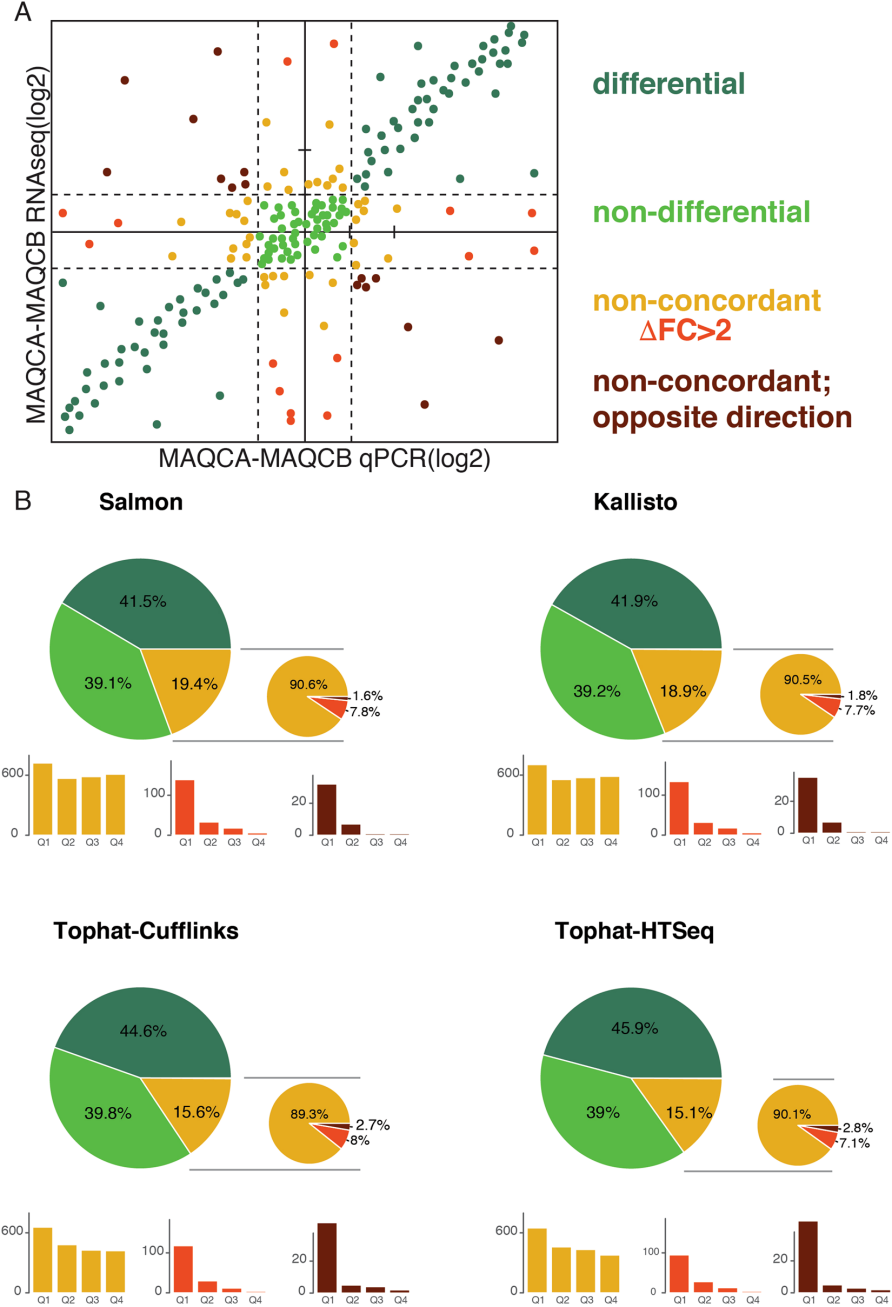
Quantification of non-concordant genes reveals that the numbers are low and similar between workflows. (**A**) A schematic overview of different classes of genes, used for further analysis, by means of a dummy example. The concordant genes between RT-qPCR and RNA-seq are either differentially expressed or non-differential for both datasets. The non-concordant genes are split into three groups, those with a ∆FC < 2, ∆FC > 2 and the ones with a FC in the opposite direction. (**B**) The percentages of genes in each of the above-described classes is shown for each workflow. For the non-concordant genes, distribution across expression quartiles (Q1 = lowest 25%) is shown. Results are based on RNA-seq data from dataset 1.

To quantify potential discrepancies between RNA-seq and qPCR, genes were divided into four groups based on their differential expression (log fold change > 1) between MAQCA and MAQCB (Fig. 4A). The first two groups consist of genes for which both methods agree on the differential expression status (i.e. differentially expressed or not differentially expressed). These genes are further referred to as concordant genes. The third and fourth group consist of genes for which both methods disagree on the differential expression status (i.e. differentially expressed by only one method or differentially expressed by both methods but with opposite direction). These genes are collectively referred to as non-concordant genes. The fraction of non-concordant genes ranged from 15.1% (Tophat-HTSeq) to 19.4% (Salmon) and was consistently lower for the alignment-based algorithms compared to the pseudoaligners (Fig. 4B). While the non-concordant fraction appears large, it mainly consists of genes for which the difference in log fold change between methods (ΔFC) is relatively low. For instance, over 66% of all genes in the non-concordant fraction have a ΔFC < 1 and 93% have a ∆FC < 2, irrespective of the workflow (Supplemental Fig. 7). We therefore defined a fifth group of genes with ΔFC > 2. These genes represent between 7.1% (Tophat-HTSeq) and 8% (Tophat-Cufflinks) of the entire non-concordant fraction (Fig. 4B) and, together with the genes that have differential expression going in opposite directions, we considered as truly deviating between RNA-seq and qPCR. When evaluating the expression levels of the various fractions of non-concordant genes, it’s clear that the non-concordant genes with ΔFC > 2 and non-concordant opposite direction genes are primarily expressed at low levels (i.e. first expression quartile, Fig. 4B and Supplemental Fig. 8). In contrast, non-concordant genes with ΔFC < 2 are equally distributed across expression quartiles (Fig. 4B). An overview of all non-concordant genes is available in Supplemental Table 2.

**Figure 5:**
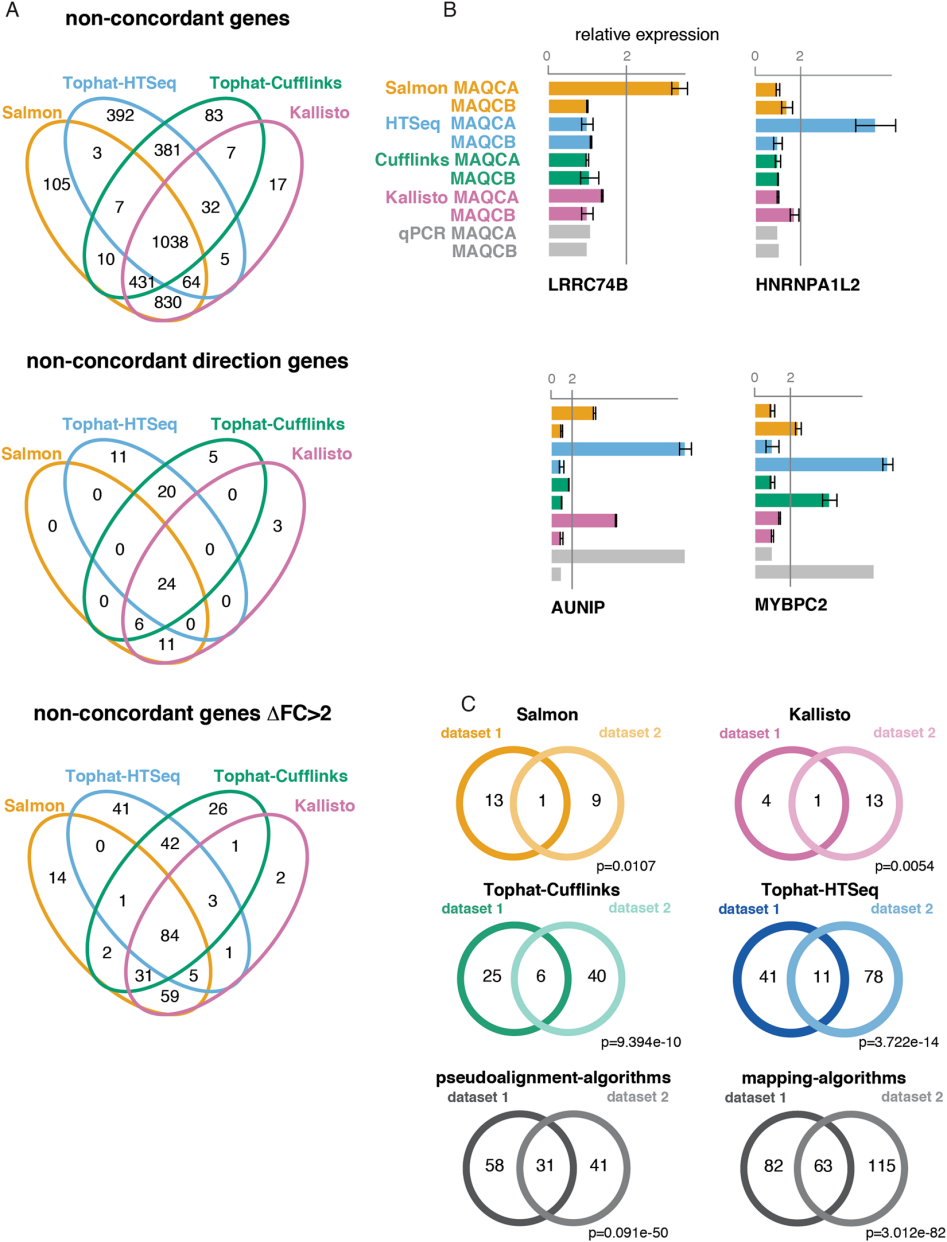
Each workflow (or workflow group) has specific non-concordant genes, which are reproducible identified in independent datasets. (**A**) Venn diagrams showing the overlap between the non-concordant genes with ∆FC < 2, non-concordant genes with ∆FC > 2 and non-concordant genes with opposite direction. (**B**) Examples of workflow-specific non-concordant genes. (**C**) Overlap of the non concordant genes with a ∆FC > 2 between two independent datasets. The p-values (Fisher Exact test) represent the significance of the overlap.

To evaluate the extent to which the non-concordant genes are workflow-specific, we assessed the overlap of non-concordant genes between workflows (Fig. 5A and Supplemental Fig. 9). While a significant number of genes are shared between all workflows, several genes were identified that are specific to one workflow or a group of workflow (i.e. alignment based and pseudoaligners). Whereas the former points to systematic discrepancies between quantification technologies (i.e. qPCR and RNA-seq), the latter points to differences between individual workflows or groups of workflows. The number of workflow-specific, non-concordant genes with ΔFC > 2 ranged from 5 (Kallisto) to 55 (Tophat-HTSeq). These are genes where the workflow fails to reproduce the differential expression (observed by qPCR and all other workflows) or genes for which the workflow observes differential expression that is not confirmed by qPCR or any of the other workflows. Examples of workflow-specific non-concordant genes with ΔFC > 2 are shown in Fig. 5B. LRRC74B and HNRNPA1L2 are differentially expressed according to Salmon and Tophat-HTSeq respectively, but are non-differential according to the other workflows and RT-qPCR. Conversely, AUNIP and MYBPC2 are non-differential according to Tophat-Cufflinks and Kallisto respectively, but differential according to RT-qPCR and the other workflows. When grouping workflows, we identified 70 non-concordant genes with ΔFC > 2 specific for pseudoalignment algorithms and 62 non-concordant genes with ΔFC > 2 specific for mapping algorithms. Similar results were obtained in the second dataset (Supplemental Figs 10–12).

To verify whether these genes were consistent between independent RNA-seq datasets, we compared results between dataset 1 and 2. Workflow-specific genes were found to be significantly overlapping between both datasets (Fig. 5C). This was especially the case for Tophat-Cufflinks and Tophat-HTSeq specific genes. Also genes specific for pseudoalignment algorithms and mapping algorithms were significantly overlapping between dataset 1 and 2 (Fig. 5B). These results suggest that each workflow (or group of workflows) consistently fails to accurately quantify a small subset of genes, at least in the samples considered for this study.

### Features of non-concordant genes

**Figure 6:**
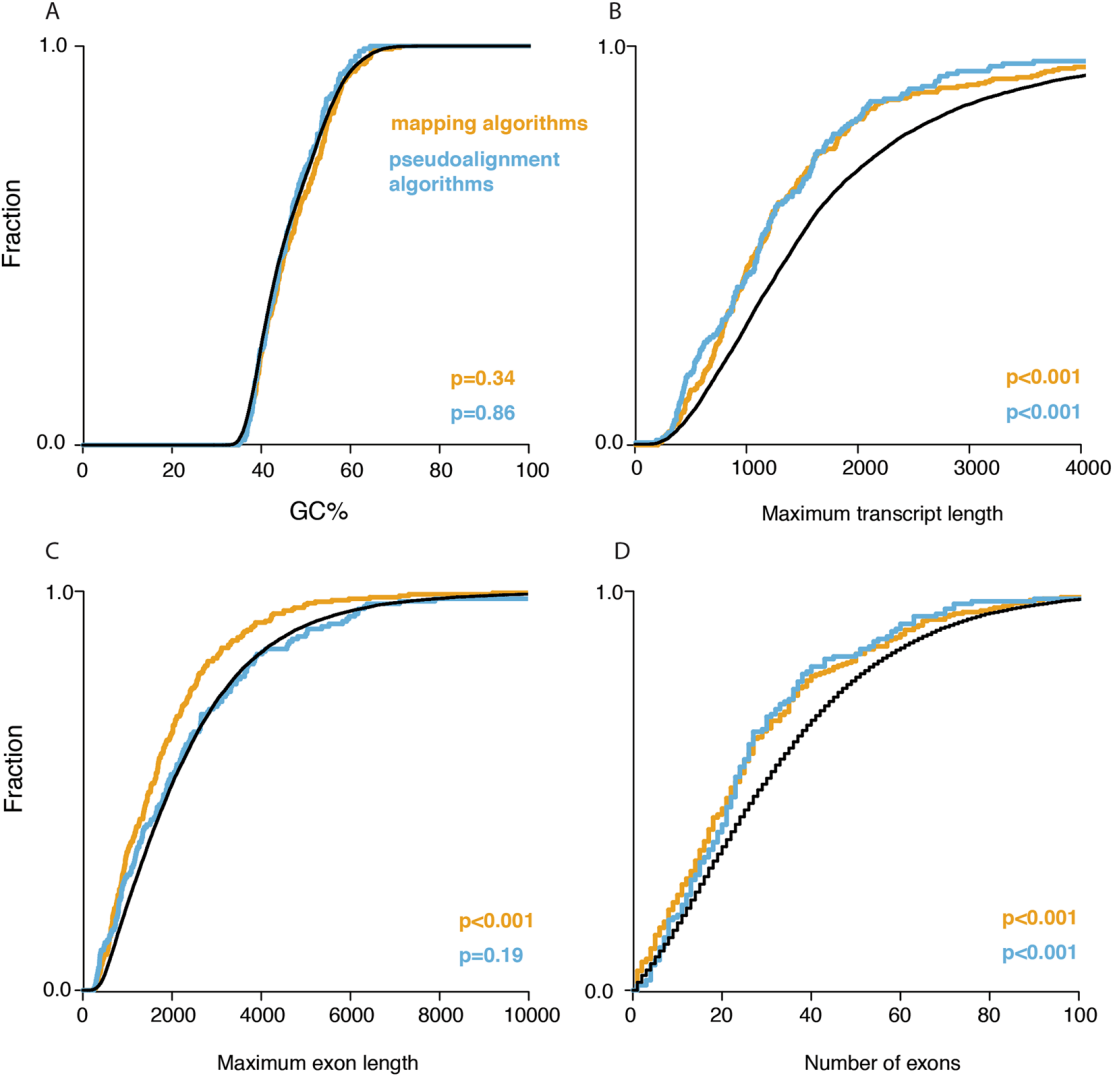
Non-concordant genes show differential characteristics compared to concordant genes. Cumulative fractions of %GC (**A**), maximum transcript length (**B**), maximum exon length (**C**) and number of exons (**D**) for concordant genes compared to non-concordant gens specific for either pseudoalignment or mapping algorithms. Kolmogorov-Smirnov p-values are indicated.

In order to evaluate why accurate quantification of specific genes failed, we computed various features including GC-content, gene length, number of exons, and number of paralogs. These features were determined for concordant and non-concordant genes and compared between both groups (Fig. 6). Non-concordant genes specific for pseudoalignment algorithms and mapping algorithms were significantly smaller (Wilcoxon: p < 0.001, Kolmogorov-Smirnov: p < 0.001) and had fewer exons (Wilcoxon: p < 0.003, Kolmogorov-Smirnov: p < 0.001) compared to concordant genes. No significant difference in GC-content or number of paralogs was observed. Besides evaluating gene characteristics, we also assessed the number of poor quality reads (below Q20) and multi-mapping reads. The number of poor quality and multi-mapping reads was higher for non-concordant compared to concordant genes. This was observed for both pseudoalignment (Chi-square: p < 2.2e-16; relative risk poor quality = 1.12, multi-mapping = 1.071) and mapping workflows (Chi-square: p < 2.2e-16; relative risk poor quality = 1.073, multi-mapping = 1.075).

## Discussion

Based on a unique dataset of RT-qPCR expression measurements for 18 080 protein-coding genes, we evaluated the performance of five RNA-seq processing workflows, including both alignment based and pseudoalignment algorithms. Of note, RNA-seq workflows not included in this study may perform differently than those selected here. We decided to run each workflow using the default analysis parameters as we reasoned that this is likely what most users do. Nevertheless, adjusting or fine-tuning these parameters might further improve performance of individual algorithms. Algorithm performance may also depend on the RNA-seq library prep method. Here, we used stranded polyA+ libraries sequenced in paired-end mode. Performance may differ when evaluating unstranded libraries, total RNA libraries or single end reads. Moreover, the annotation of the reference transcriptome could also influence quantification results. RT-qPCR assays may for instance also detect transcripts not included in the reference annotation and hence not taken into account by the RNA-seq processing workflows. This could result in an underestimation of the TPM values with respect to Cq-values obtained by qPCR. However, the expression correlation plots indicate that more genes show the opposite pattern and have a higher expression when quantified by RNA-seq as compared to RT-qPCR (Fig. 1). This may, in part, be explained by differences in amplification efficiency. Another possible explanation is that for this benchmark a transcriptome, filtered for transcripts detected by the qPCR assays, was used. Reads mapping to shared exons from transcripts not detected by the qPCR assay are therefore expected to increasing the quantification values for the RNA-seq workflows. Using a pre-filtered transcriptome indeed results in higher gene-level TPM-values for a small subset of genes compared to a non-filtered transcriptome, where gene-level TPM-values were generated by summing transcript-level TPM-values of transcripts detected by the qPCR assays (Supplemental Fig. 13). Fold changes between samples were largely unaffected. Taken together, the use of an extensive or non-filtered annotation will result in more reliable quantification. For the HTSeq workflow, post-quantification filtering is not possible, resulting in a lower correlation with RT-qPCR data. Of note, this phenomenon is due to the transcript specificity of the RT-qPCR assay designs and not to the quantification workflow itself. Another caveat of using a filtered transcriptome is that increased TPM-values of some genes will result in decreased TPM-values of others given the relative nature of this measure. However, this should not affect any of the analysis where differences between samples are compared.

For the comparison between RNA-seq and RT-qPCR, we focussed our analysis on differential gene expression correlations as these are conceptually more relevant and more closely resemble the main application of RNA-seq. We deliberately avoided introducing differential gene expression algorithms like DESeq^15^, edgeR^16^ or LimmaVoom^17^ as these may further influence the results and prevent us from assessing workflow differences at the level of gene expression quantification. Instead, differential gene expression was assessed by means of fold change correlations directly derived from TPM values. From these analyses, we concluded that the choice of mapper hardly affects results and that, in general, there is a high concordance between RT-qPCR and RNA-seq for each of the RNA-seq processing workflows. This is exemplified by the high number of genes (80–85%) for which a concordant (differential or non-differential) gene expression was observed. These conclusions are in contrast to those published by Teng *et al*. who reported a poor performance of RNA-seq processing algorithms when evaluating differential gene expression^13^. This may be due to the fact that conclusions in this study were partially based on simulated data. Performance was indeed higher when, in the same study, microarray data was used to benchmark the RNA-seq results.

As the non-concordant genes in our study were mostly borderline, we defined a set of severely non-concordant genes for which fold changes differed substantially between RNA-seq and RT-qPCR. These genes represented on average 1.8% of the total number of genes considered (n = 13 045) and were reproducibly identified between datasets. This implicates that both alignment and pseudoalignment algorithms have problems with a limited but specific set of genes. These genes were typically lower expressed, smaller and had fewer exons, confirming findings from a recent study reporting on problematic genes in RNA-seq data^18^. In addition, the reads mapping to non-concordant genes had lower quality and mapped more often to multiple regions. Although the effects of the individual features (i.e. transcript length, number of exons and read quality) are small, combinations of these features may better explain the non-concordance of individual genes. However, additional features that were not assessed here may also contribute. Whether the same genes will pose problems in samples other than those assessed in this study requires further examination. Finally, we cannot exclude the possibility that the 18080 protein-coding genes considered here have features that favour accurate quantification by RNA-seq, compared to genes not included in this study. For these genes, primer design is likely to be hampered by specificity issues. Such genes would also be more challenging to analyse using RNA-seq. Therefore, it remains to be determined to what extent our findings can be extrapolated to all genes (i.e. protein coding genes not included in the study and long non-coding RNAs).

## Conclusion

All workflows show a good concordance with RT-qPCR expression measurements and no workflow outperforms the others. Of note, each workflow revealed a small but specific set of genes with inconsistent expression measurements, reproducibly identified in independent datasets. These genes were typically smaller, had fewer exons and were lower expressed compared to genes with consistent expression measurements. Careful validation is warranted when evaluating RNA-seq based expression profiles for this specific set of genes.

## Methods

### Samples

For this benchmark we used the well-characterized MAQC-I RNA-samples MAQCA (Universal Human Reference RNA, Agilent Technologies,) and MAQCB (Human Brain Reference RNA, Thermo Fisher Scientific)^14^. For both samples, RNA-sequencing was performed.

### RT-qPCR

RT-qPCR data for 18080 protein-coding genes were generated in the context of the Sequencing Quality Control study (SEQC) (17) using PrimePCR assays (BioRad) (Supplemental Table 1). In order to define the ensemble of transcripts amplified by every individual qPCR assay, assays were re-mapped on the reference transcriptome (ensembl v75). Genes with a Cq-value between 11 and 32 were considered for further analysis. Cq-values were normalized using the global mean normalization strategy^19^.

### RNA-Seq

For the first RNA-seq dataset (GSE83402), we generated replicate libraries for MAQCA and MAQCB using the stranded TruSeq mRNA library prep kit (Illumina) with 100 ng input RNA according to the manufacturer’s instructions. Libraries were sequenced on a NextSeq 500 (Illumina), generating paired-end 75 bp reads, with a mean of 50 M reads per sample. A second, independent RNA-seq dataset for MAQCA and MAQCB was obtained from the the SEQC study (GSE47792)^20^. Two replicates for MAQCA (ILM_BGI_A_1 and ILM_BGI_A_2) and MAQCB (ILM_BGI_B_1 and ILM_BGI_B_2), sequenced at the Beijing Genomics Institute with a mean of 73 M reads, were selected.

### RNA-seq data processing

Fastq files were processed with five popular workflows (Tophat-HTSeq, Tophat-Cufflinks, STAR-HTSeq, Kallisto and Salmon) using the most recent versions of the software available at the time of analysis (Bowtie2 v2.1.0, Tophat v2.0.10, Cufflinks v2.1.1, HTSeq v0.5.4, Kallisto v0.42.1 and Salmon v0.6.0). For every workflow, default analysis settings and parameters were used. The same reference transcriptome was used for all workflows (Ensembl GRCh37, release 75). For Tophat-Cufflinks and Tophat-HTSeq, the transcriptome was filtered for transcripts detected by the RT-qPCR assays prior to running the Cufflinks and HT-seq algorithms. For Salmon and Kallisto the quantification was performed on the full transcriptome and gene-level TPM-values were calculated by summing transcript-level TPM values of those transcripts detected by the RT-qPCR assays. Tophat mapped on average 77.2% of the reads. For Tophat-Cufflinks and Tophat-HTSeq, the FPKM values were converted to TPM^21^. To get the TPM values for Tophat-HTSeq and Star-HTSeq, we took into account the length of the longest transcript. Calculating TPM-values using the median or minimum transcript length did not change fold-change correlation, however for the absolute values a higher correlation was obtained by using the length of the longest transcript (Supplemental Fig. 14). To define a TPM cutoff, we applied a measure published previously in the miRNA Quality Control Study^22^, relying on single positive reduction in replicate experiments. We defined this cut-off for both datasets, for both samples and for all workflows (Supplemental Fig. 15). Based on these values, one general cut-off was defined. As genes were also filtered based on the qPCR expression data, we decided to set this cut-off just below the lowest cut-off that was calculated, at 0.1 TPM. The cut-off was applied as such that only those genes were retained with expression above 0.1 TPM for all workflows and samples. The fastq files of the first dataset and output of the different workflows are available trough GEO (GSE83402).

1 $${TPM}=\,(\frac{{FPK}{{M}}_{i}}{{\sum }_{j}{FPK}{{M}}_{j}})\cdot {10}^{6}$$

### Statistics

For the statistical analysis, R (version 3.2.2) was used. Expression correlation was calculated using either Pearson or Spearman. To test for significant overlap of individual elements in the Venn diagrams, either the Fisher Exact test, for 2 sets, of the Super Exact test^23^, for multiple sets, was used. To test differences between sets of genes, the non-parametric Wilcoxon signed-rank test and the Kolmogorov-Smirnov test were used.

## Electronic supplementary material

Supplemental Figures

Supplemental Table1

Supplemental Table2
