# Supplementary material for: Benchmarking of RNA-sequencing analysis workflows using whole-transcriptome RT-qPCR expression data
Source: Sci Rep. 2017 May 8;7:1559. doi: 10.1038/s41598-017-01617-3 (PMC5431503; doi:10.1038/s41598-017-01617-3)
Supplement: Supplementary file 1 — Supplemental Figures [file 41598_2017_1617_MOESM1_ESM.pdf]

Benchmarking of RNA-sequencing analysis workflows using whole-transcriptome RT-qPCR expression data

Celine Everaert[1,2,3], Manuel Luypaert[4], Jesper L.V. Maag[5], Quek Xiu Cheng[5], Marcel E. Dinger[5], Jan Hellemans[4] and Pieter Mestdagh[1,2,3,*]

[1] Center for Medical Genetics, Ghent University, Ghent, Belgium

[2] Cancer Research Institute Ghent, Ghent University, Ghent, Belgium

[3] Bioinformatics Institute Ghent N2N, Ghent University, Ghent, Belgium

[4] Biogazelle, Ghent, Belgium

[5] Kinghorn Cancer Center, Sydney, Australia

* To whom correspondence should be addressed. Tel: +32 9 332 6979; Fax: +32 9 332 6549; Email: pieter.mestdagh@ugent.be

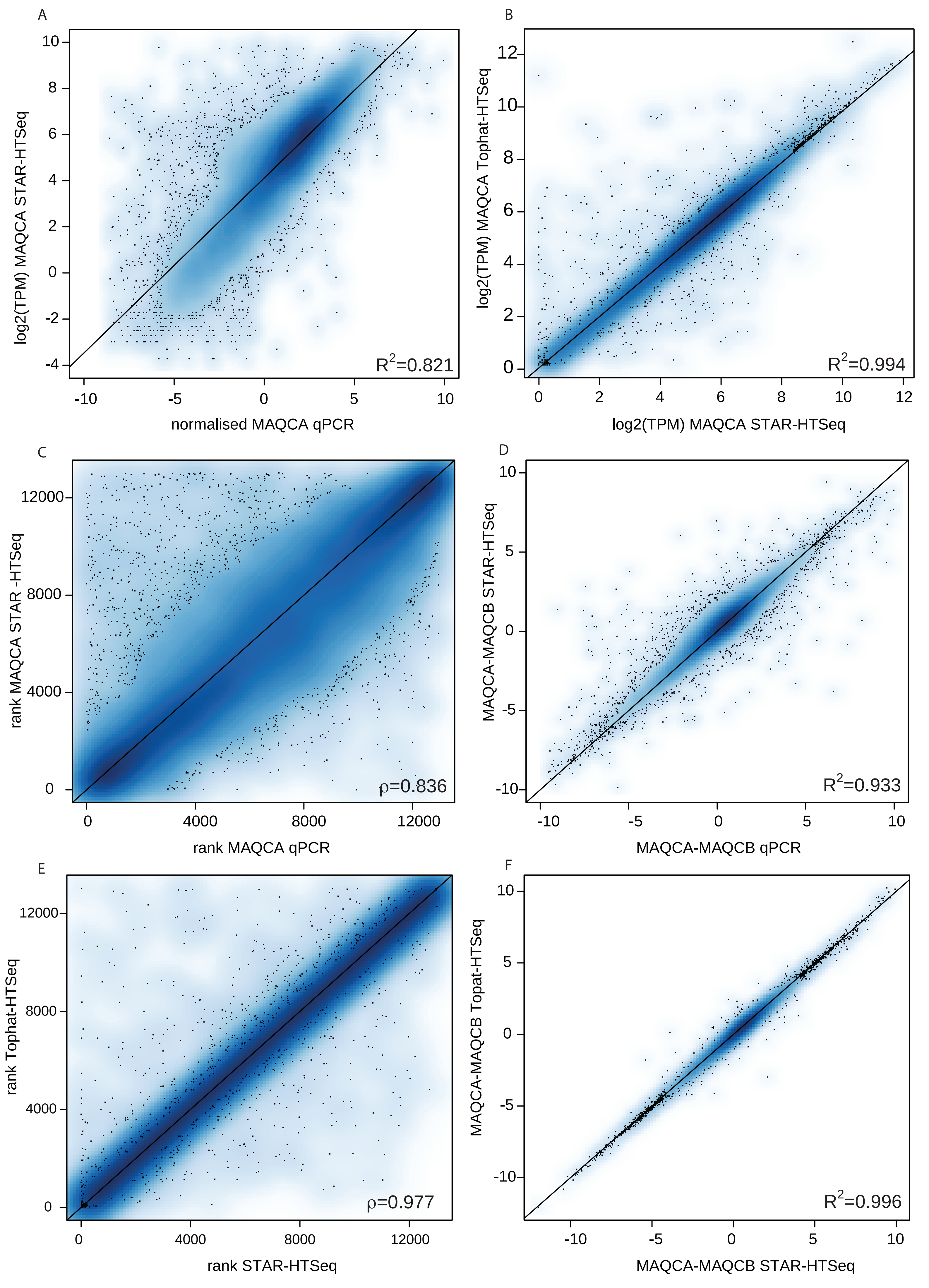
A
log2(TPM) MAQCA STAR-HTSeq
normalised MAQCA qPCR
R2=0.821
B
log2(TPM) MAQCA Tophat-HTSeq
log2(TPM) MAQCA STAR-HTSeq
R2=0.994
C
rank MAQCA STAR -HTSeq
rank MAQCA qPCR
ρ=0.836
D
MAQCA-MAQCB STAR-HTSeq
MAQCA-MAQCB qPCR
R2=0.933
E
rank Tophat-HTSeq
rank STAR-HTSeq
ρ=0.977
F
MAQCA-MAQCB Topat-HTSeq
MAQCA-MAQCB STAR-HTSeq
R2=0.996

Supplemental Figure 1 The choice of alignment algorithm has no effect on the performance of the workflow. Abundance (a), Rank (c) and fold change correlation (e) between qPCR and the STAR-HTSeq workflow. Abundance (b), Rank (d) and fold change correlation (f) between STAR-HTseq and Tophat-HTSeq. The correlation of the fold changes was calculated by the Pearson correlation coefficient. The rank correlation was calculated using Spearman’s Rank.

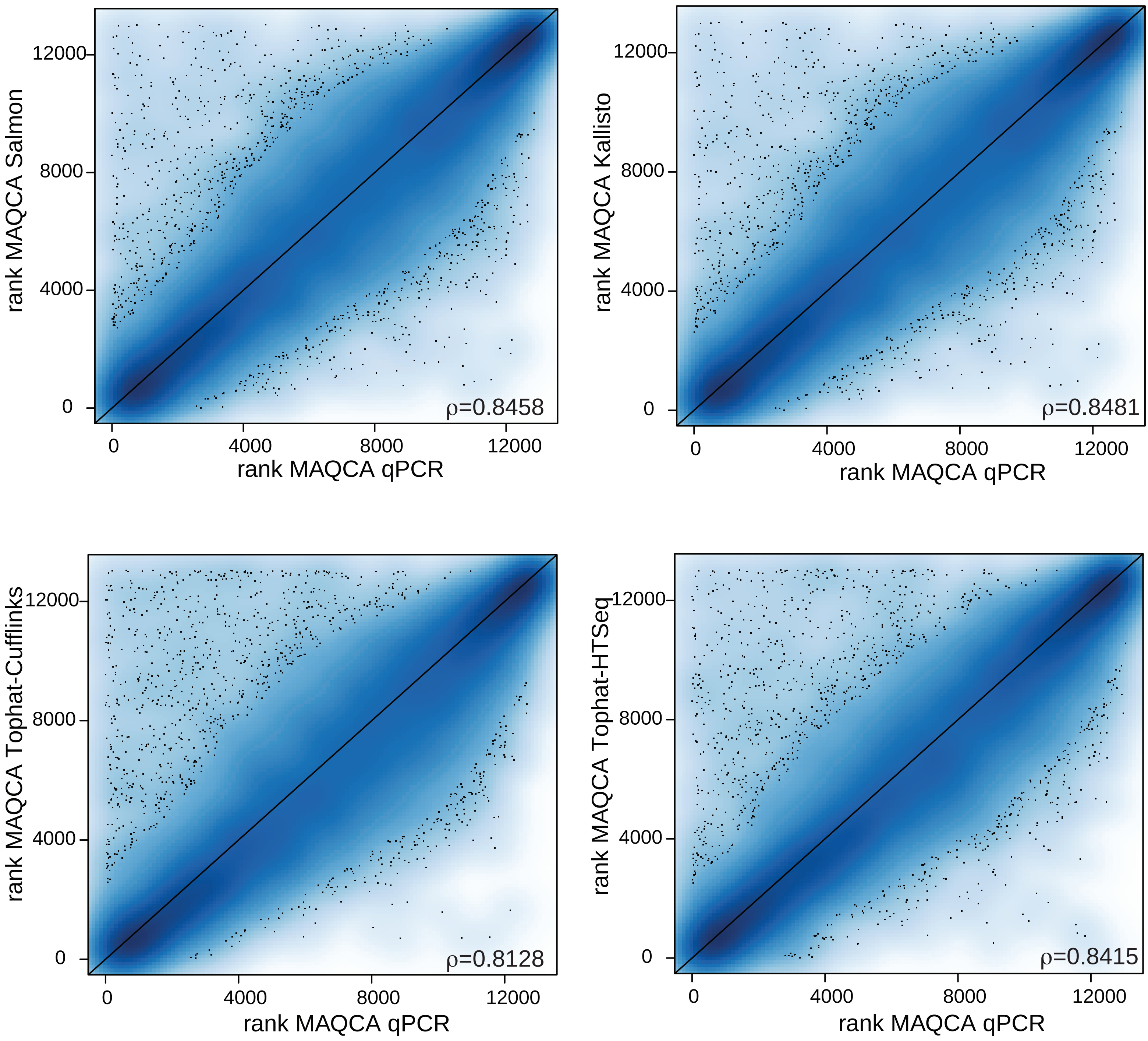

Supplemental Figure 2 Each workflow has a high rank correlation between RT-qPCR and RNA-seq data. Spearman's Rank correlation coefficients are indicated. Results are based on RNA-seq data from dataset 1.

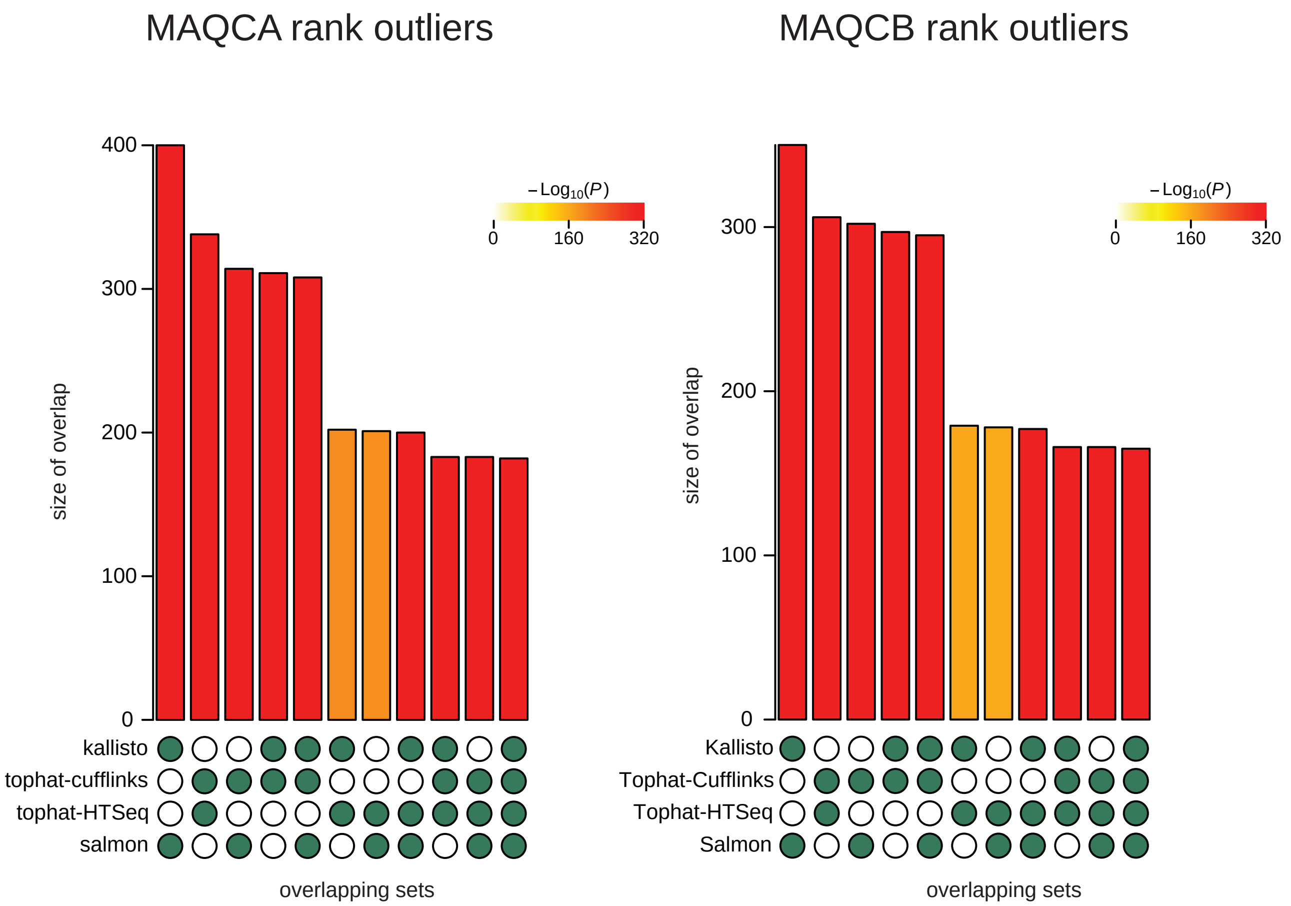

Supplemental Figure 3 The Super Exact test shows a significant overlap between the rank outliers genes for MAQCA as MAQCB.

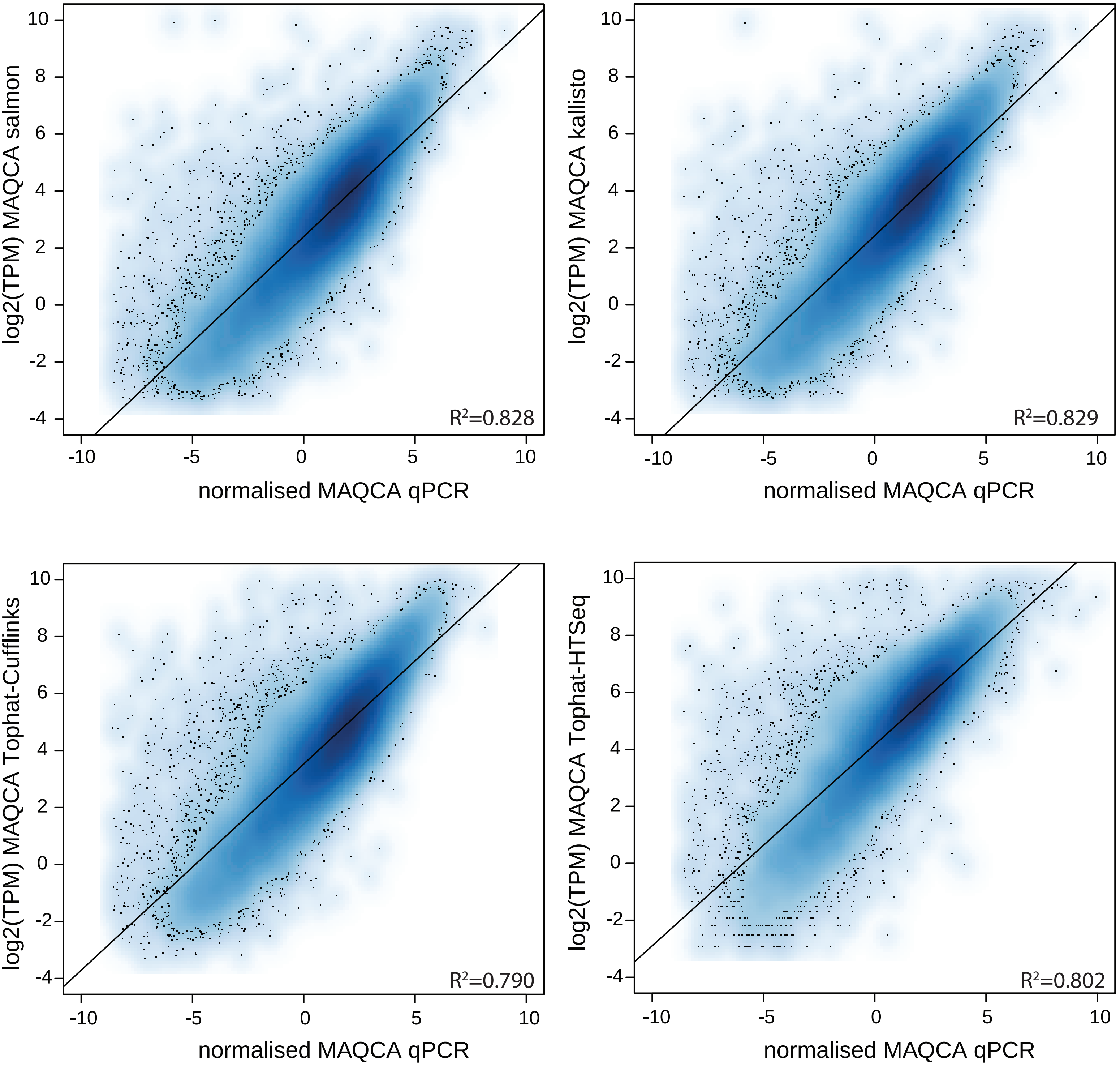

Supplemental Figure 4. Each workflow has a high abundance correlation between RT-qPCR and RNA-seq data. Pearson correlation coefficients are indicated. Results are based on RNA-seq data from dataset 2.

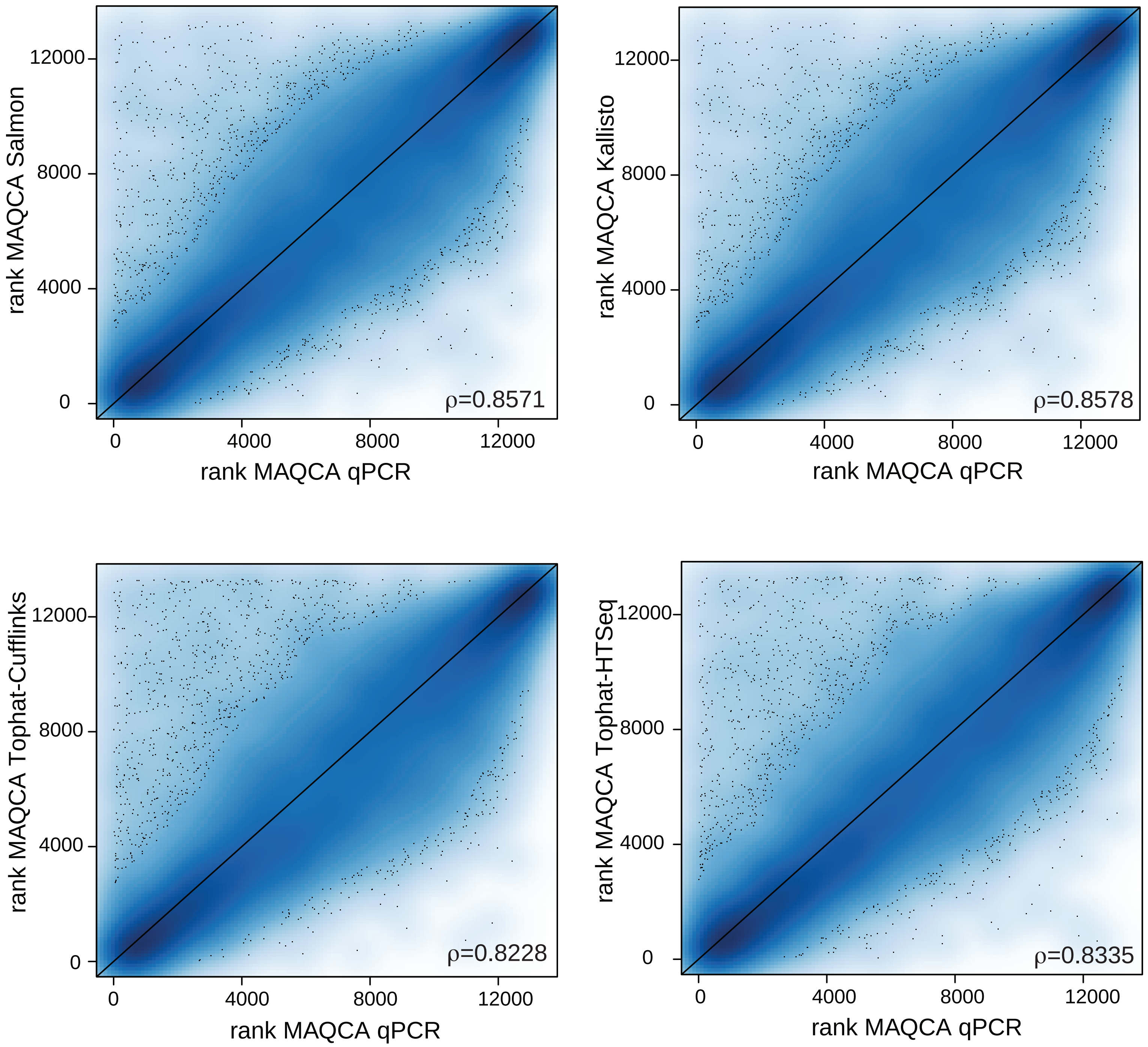

Supplemental Figure 5. Each workflow has a high rank correlation between RT-qPCR and RNA-seq data. Spearman's Rank correlation coefficients are indicated. Results are based on RNA-seq data from dataset 2.

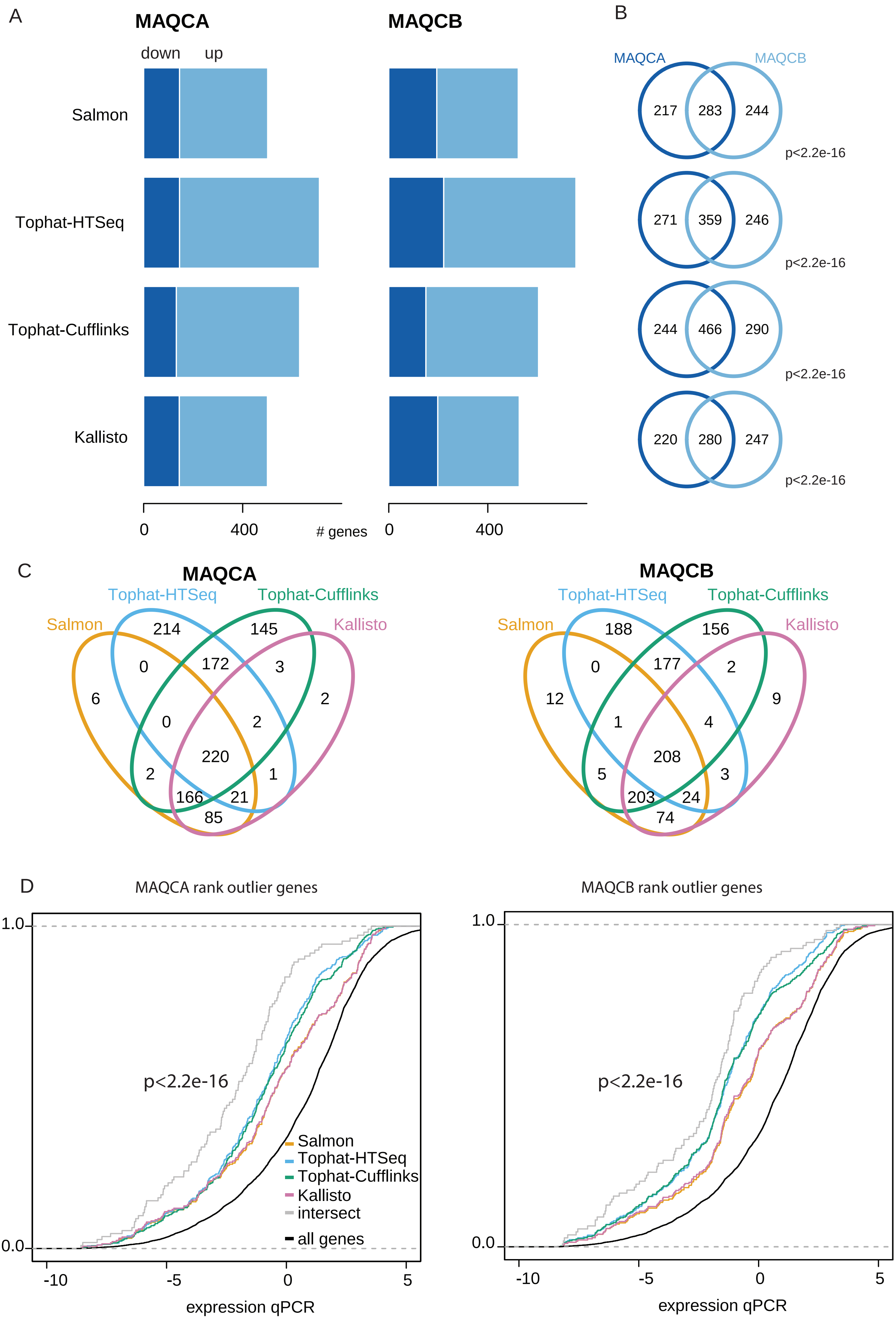
A
MAQCA
MAQCB
down
up
Salmon
Tophat-HTSeq
Tophat-Cufflinks
Kallisto
0
400
genes
0
400
B
MAQCA
MAQCB
217 283 244
p<2.2e-16
271 359 246
p<2.2e-16
244 466 290
p<2.2e-16
220 280 247
p<2.2e-16
C
MAQCA
Salmon
Tophat-HTSeq
Tophat-Cufflinks
Kallisto
214 145 0 172 3 6 2 0 2 220 2 1 166 21 85
MAQCB
Salmon
Tophat-HTSeq
Tophat-Cufflinks
Kallisto
188 156 0 177 2 12 9 1 4 208 5 3 203 24 74
D
MAQCA rank outlier genes
1.0
0.0
p<2.2e-16
Salmon
Tophat-HTSeq
Tophat-Cufflinks
Kallisto
intersect
all genes
-10 -5 0 5
expression qPCR
MAQCB rank outlier genes
1.0
0.0
p<2.2e-16
-10 -5 0 5
expression qPCR

Supplemental Figure 6. The overlap of the rank outlier genes between samples (MAQCA and MAQCB) and workflows is significant. (A) The number of genes with an (absolute) rank shift of more than 5000 are indicated. Genes marked as down have a higher expression rank in RT-qPCR, genes marked as up have a higher expression rank in RNA-seq. (B) The overlap of genes with an absolute rank shift of more than 5000 between MAQCA and MAQCB is significant for each workflow (Fisher exact test) (C) The overlap of the genes with an absolute rank shift of more than 5000 between the different workflows is significant (Super exact test). (D) Genes with an absolute rank shift of more than 5000 have an overall lower expression. The Kolmogorov-Smirnov p-value for the intersection of rank outlier genes between methods is shown. Results are based on RNA-seq data from dataset 2.

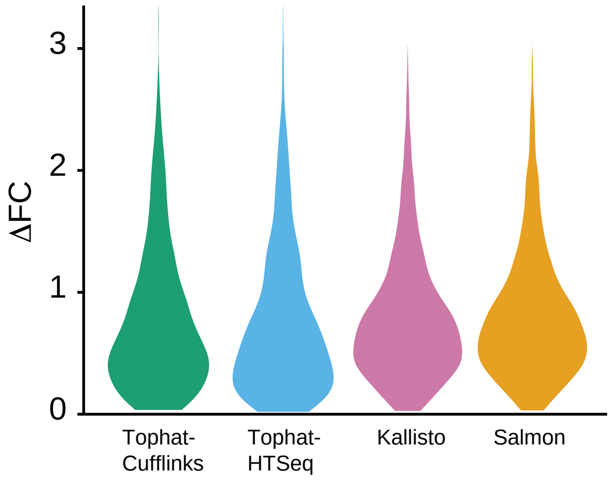

Supplemental Figure 7 Violin plots showing that most genes have a low absolute difference in fold change between RT-qPCR and RNA-seq.

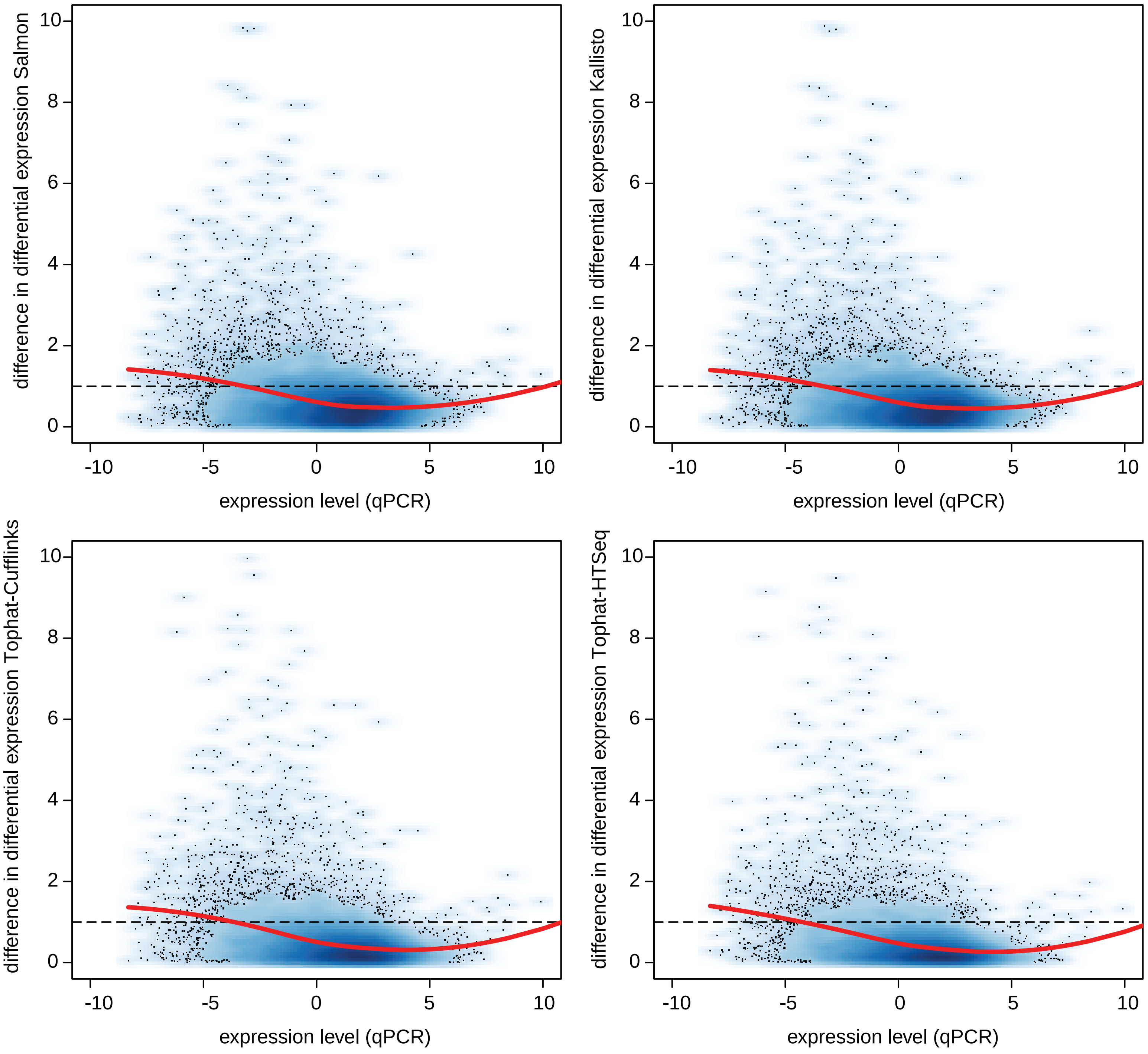

Supplemental Figure 8 Genes with lower expression levels (as measured by RT-qPCR) show a bigger difference in differential expression (ΔFC) when comparing RNA-seq and RT-qPCR.

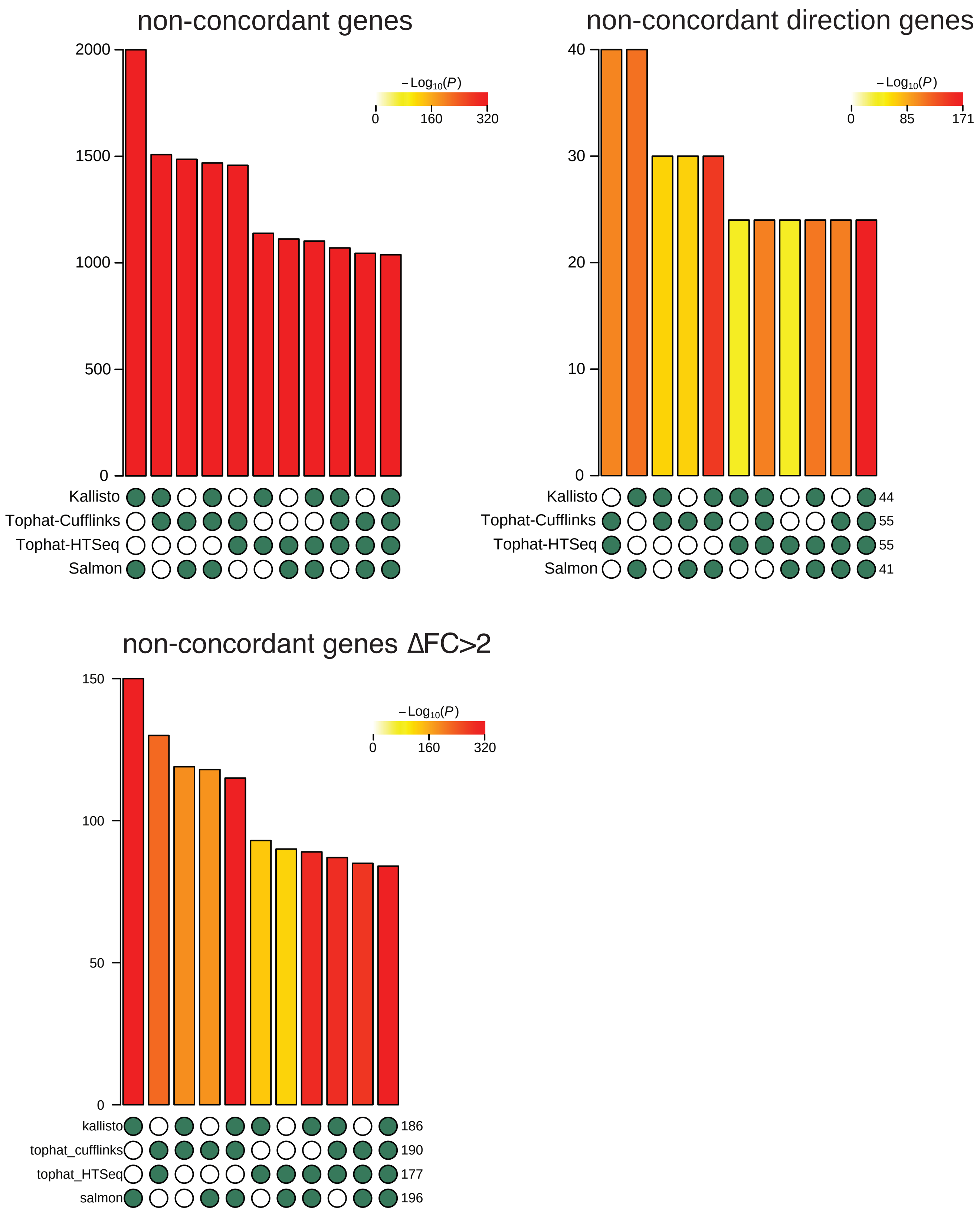

Supplemental Figure 9 The Super Exact test shows a significant overlap between the non-concordant genes with ΔFC < 2, non-concordant genes with ΔFC > 2 and non-concordant genes with opposite direction.

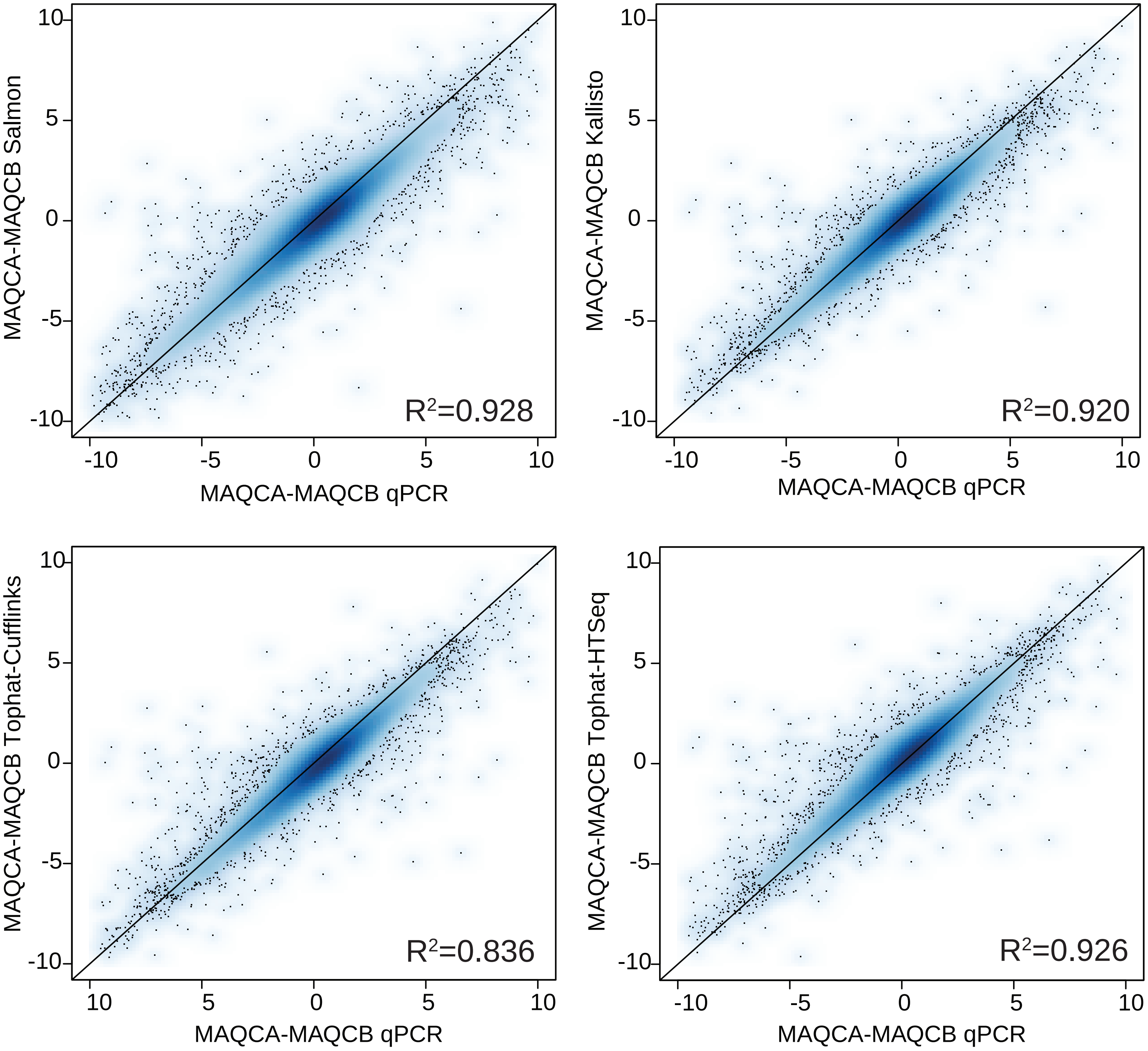

Supplemental Figure 10 High fold change correlation between RT-qPCR and RNA-seq data for each workflow. The correlation of the fold changes was calculated by the Pearson correlation coefficient. Results are based on RNA-seq data from dataset 2.

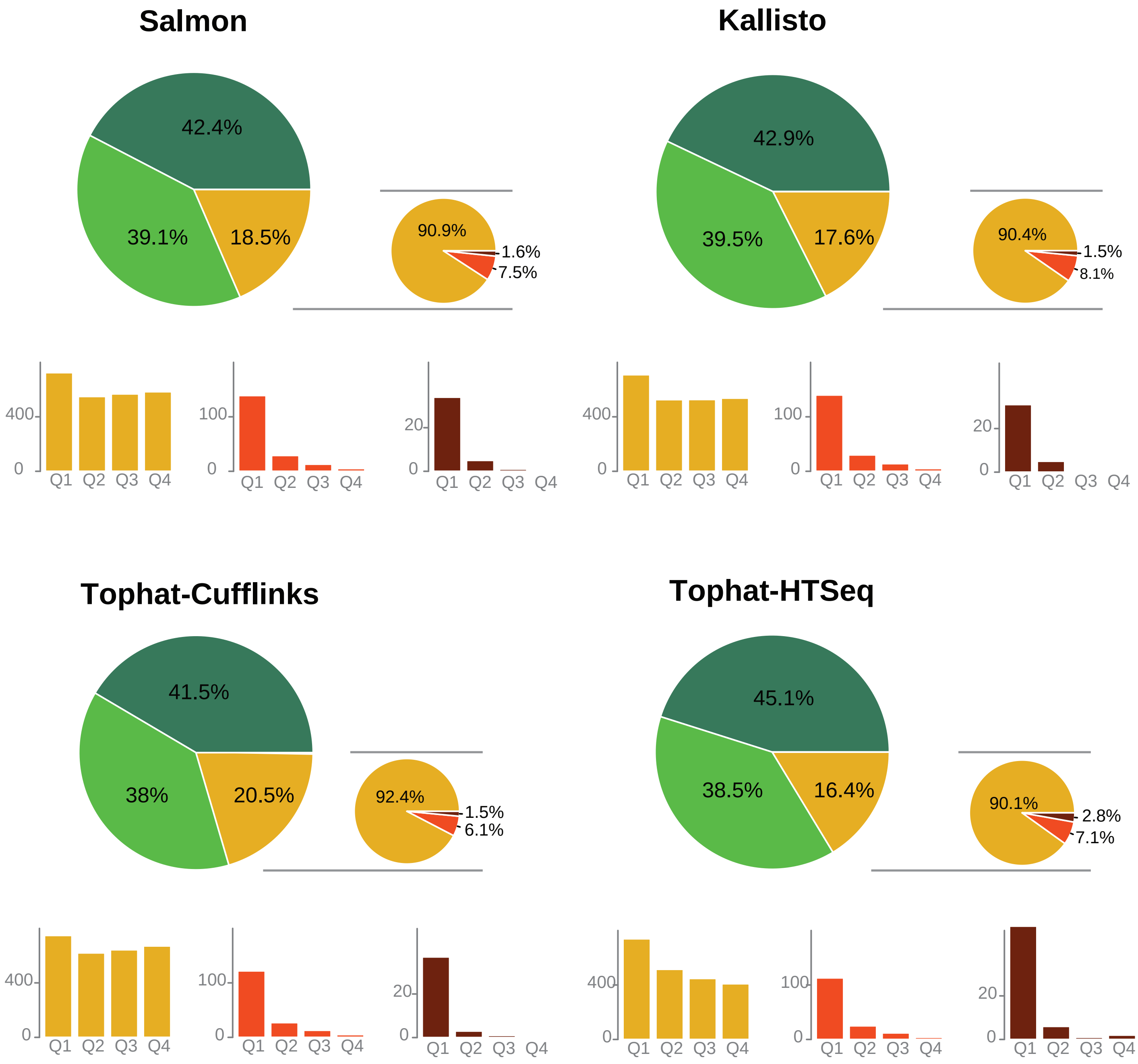

Supplemental Figure 11. Quantification of non-concordant genes reveals that the numbers are low and similar between workflows. (A) A schematic overview of different classes of genes, used for further analysis, by means of a dummy example. The concordant genes between RT-qPCR and RNA-seq are either differentially expressed or non-differential for both datasets. The non-concordant genes are split into three groups, those with a ΔFC < 2, ΔFC > 2 and the ones with a FC in the opposite direction. (B) The percentages of genes in each of the above described classes is shown for each workflow. For the non-concordant genes, distribution across expression quartiles (Q1= lowest 25%) is shown. Results are based on RNA-seq data from dataset 2.

non-concordant genes

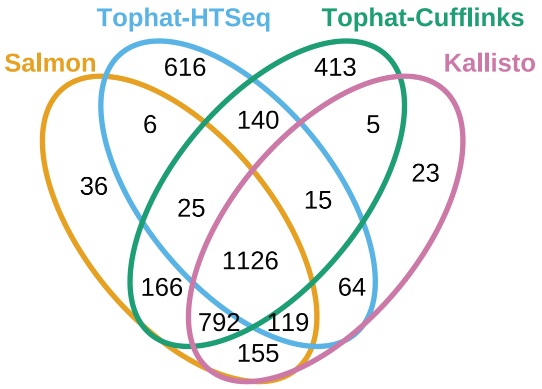

non-concordant direction genes

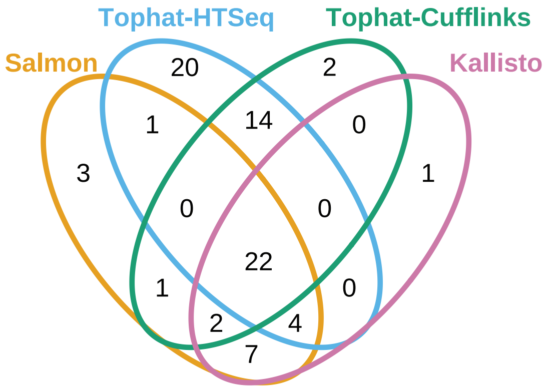

non-concordant genes ΔFC>2

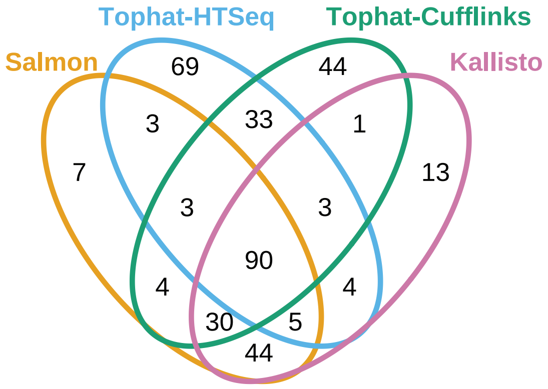

Supplemental Figure 12. Each workflow (or workflow group) has specific non-concordant genes. Venn diagrams showing the overlap between the non-concordant genes with ΔFC < 2, non-concordant genes with ΔFC > 2 and non-concordant genes with opposite direction.

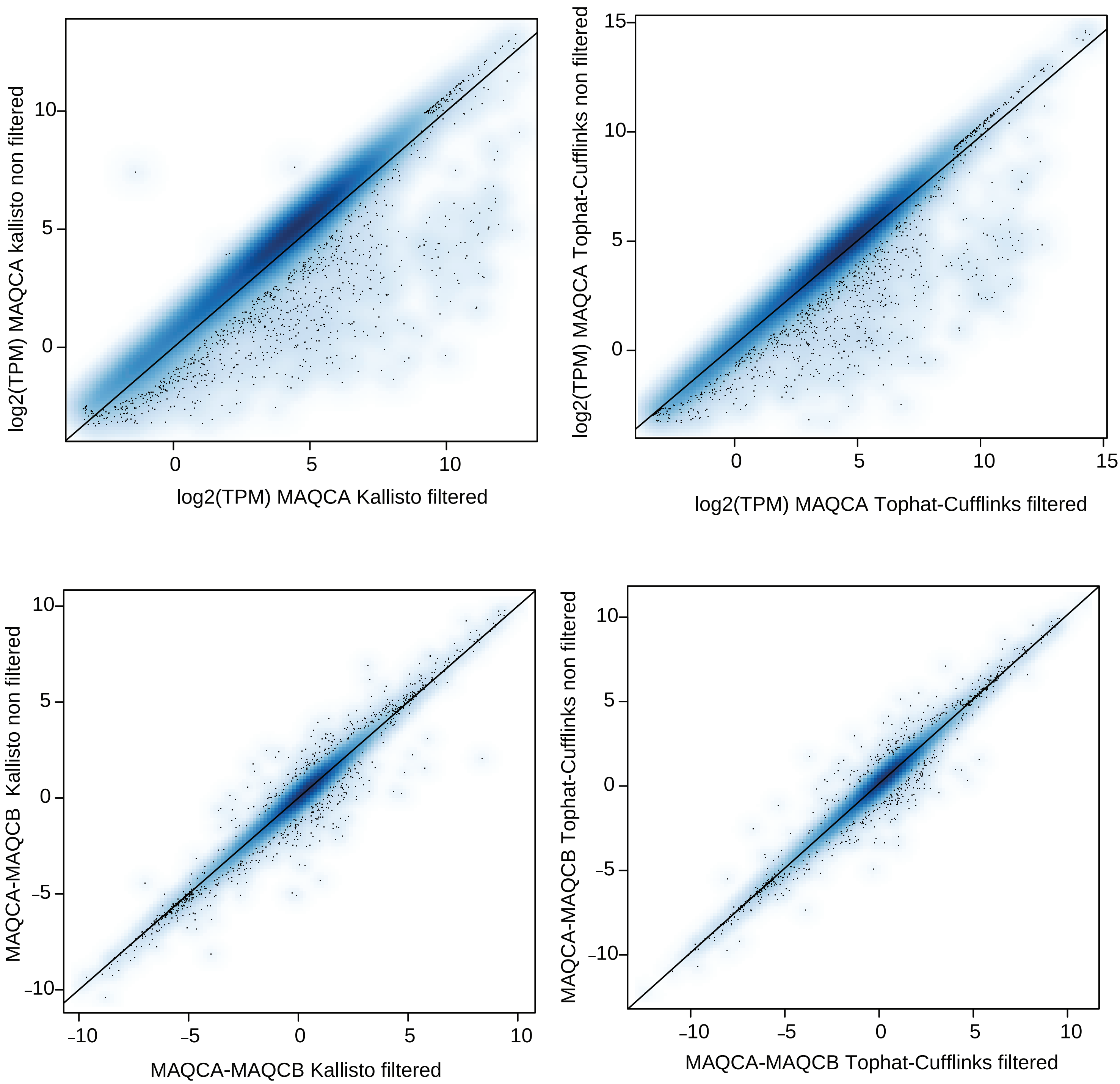

Supplemental Figure 13. Comparison between the absolute expression values and fold changes of MAQCA (and MAQCB) quantified by Kallisto and Cufflinks using a full or filtered transcriptome

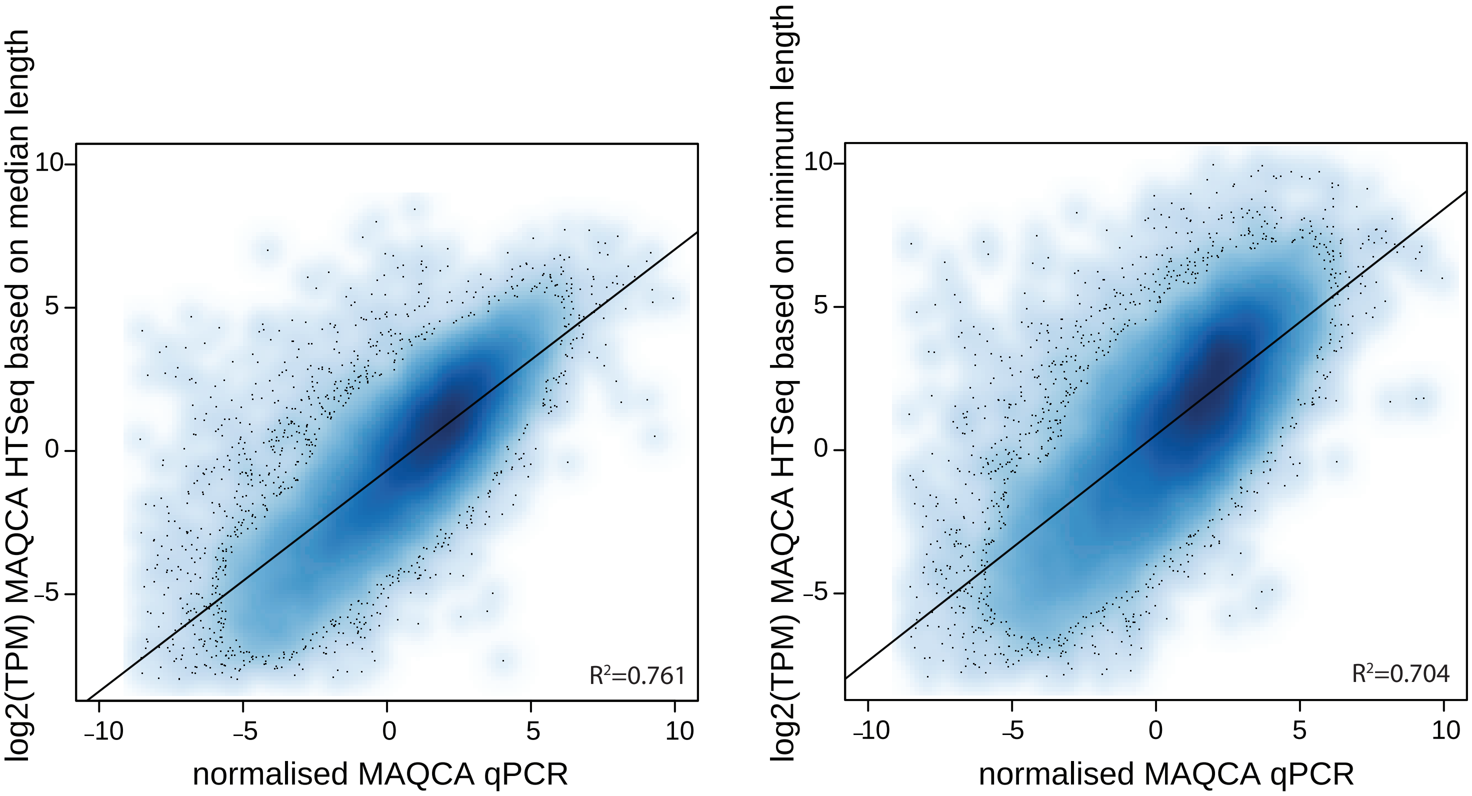

Supplemental Figure 14. Conversion from counts to TPM by median and minimum length of transcripts resulted in a Pearson correlation coefficient of 0.761 and 0.704 respectively.

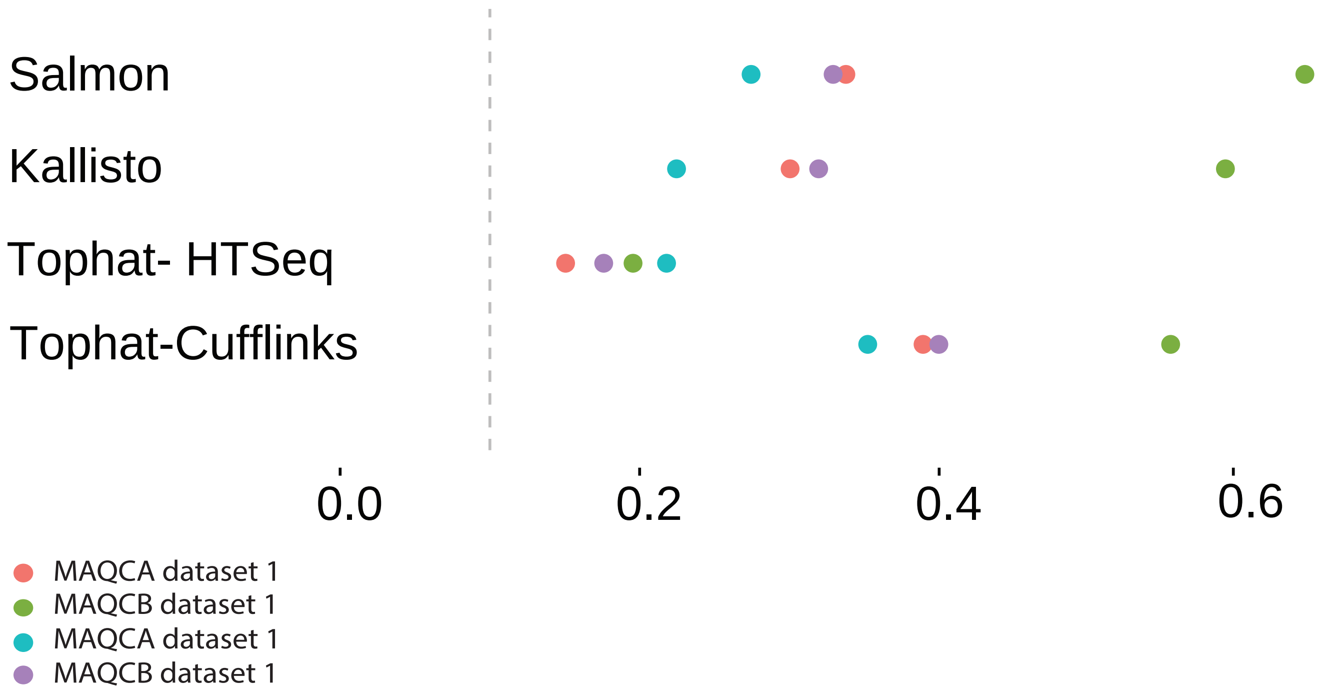

Supplemental Figure 15. Defining a TPM cut-off, based on single positive reduction in replicates.
